# In situ synthesis of silver nanoparticles on the surface of PDMS with high antibacterial activity and biosafety toward an implantable medical device

**DOI:** 10.1186/s40580-017-0126-x

**Published:** 2017-11-27

**Authors:** Joong Hyun Kim, HyeungWoo Park, Soo Won Seo

**Affiliations:** Medical Device Development Center, Daegu-Gyeongbuk Medical Innovation Foundation, Daegu, 701-310 South Korea

**Keywords:** Antibacterial medical device, Silver nanoparticles, Silicone, Catheter, Oxygen plasma

## Abstract

We developed a straightforward method to fabricate antibacterial silicon films via the in situ synthesis of silver nanoparticles (AgNPs) on a polydimethylsiloxane (PDMS) film. To grow AgNPs attached on the film, AgNP seeds were synthesized through the reduction of silver ions electrostatically bound to hydroxyl groups formed on the surface of the film after treatment with air plasma. In the growth reaction, silver ions were reduced on the seeds of AgNPs by sodium citrate in a solution of AgNO_3_, which allowed for the formation of AgNPs with sizes of up to ~ 500 nm, which The formed AgNPs on the films were characterized using UV–vis spectrophotometer, scattering electron microscope and induced coupled mass spectrometer. The amount of AgNPs was estimated to be less than 0.05% of the total film weight. Even though it was coated with a small amount of AgNPs, the PDMS film exhibited reduction of *E. coli* and *S. aureus* with values of log_10_ 4.8 and log_10_ 5.7, respectively. The biosafety of the AgNP-attached PDMS film was examined by contact of cells with the film or film eluent. Counting of viable cells revealed no significant cytotoxicity of the in situ-fabricated AgNPs on the PDMS film.

## Introduction

Since the first use of a silicone elastomer tube for duct repair in biliary surgery 60 years ago, silicone materials have been widely used in medical applications because of their superior biocompatibility compared with that of other materials [1]. Notably, PDMS-based elastomers satisfy the criteria for biocompatibility, including nonirritating and nonsensitizing behaviors. Additionally, PDMS is thermally and chemically stable. These characteristics have allowed for the long-term use of PDMS-based implants [2, 3]. However, the hydrophobic nature of PDMS results in the adhesion of bacteria on the surface of these devices, leading to microbial infections. In the United States, more than 250,000 primary blood stream infections occur annually among 150 million intravascular devices [4]. Antibacterial properties of medical devices can be achieved by coating the surface or mixing a PDMS elastomer with biocides, such as antibiotics, silver ion, furanones, or 3-(trimethoxysilyl)-ropyldimethyloctadecylammonium chloride [5]. Among reported bacterial killing agents, silver nanoparticles (AgNPs) are attractive because they show unique properties such as long-term stability and broad antibacterial activities [6]. AgNP—coated implantable medical devices such as catheters, prosthetics, bone adhesives, contact lenses, and ureteral stents have shown promising results that support the use of AgNPs as potential antibacterial agents in such devices [7–10]. Several methods to fabricate polymer–nanoparticle composites have been reported, such as mixing AgNPs with elastomers [11], immobilization of AgNPs on the surface of polymers [12], and synthesizing AgNPs inside of elastomers [13]. The silver ions released from AgNPs play key roles in such systems demonstrating antibacterial effects. Since most AgNPs mixed with elastomers should not be exposed to the external environment, a small number of mixed AgNPs were used as antibacterial agents. Therefore, immobilizing methods are more effective than mixing methods. However, immobilizing methods are more complex and expensive than mixing methods because of the additional conjugating or layer-by-layer chemistry between the surface of polymers and AgNPs [11, 12]. Herein, we present a method to synthesize AgNPs on the surface of PDMS. An easy and facile process for the in situ fabrication of nanoparticles with no further conjugating chemistry for the polymer or nanoparticle is reported. Effective antibacterial activity of the nanocomposites against both *E. coli and S. aureus* was achieved. Additionally, no cellular toxicity of the nanocomposites was observed.

## Experimental

Silver nitrate (AgNO_3_, 99%), sodium hydroxide, hydrochloric acid, sodium chloride, yeast extract, d-glucose, meat extract, Mueller–Hinton broth (MHB), 3-(4,5-dimethylthiazol-2-yl)-2,5-diphenyltetrazoliumbromide (MTT), and sodium citrate (99%) were purchased from Sigma-Aldrich. Polydimethylsiloxane (MED-664) was purchased from Nusil. Acetone, isopropanol, and ethanol were obtained from Merck. Peptone, beef extract agar powder, Eagle’s minimum essential medium (EMEM), and tryptone were purchased form BD Difco. Fetal bovine serum (FBS) was purchased from Fisher Scientific. For antibacterial tests, *E. coli* (ATCC 8739) and *S. aureus* (ATCC 6538P) were used. Cytotoxicity effects mediated by the PDMS film were tested using fibroblast cells.

The PDMS film was fabricated according to the manufacturer’s instructions. In brief, part A and B of the kit were mixed thoroughly at a ratio of 1:1 by weight using an up-stirrer (IKA), and the mixture was then degassed under vacuum to remove any entrapped air bubble. The homogeneous mixture was uniformly dispersed on a Teflon film coated plate, and then was cured at 60 °C for 16–18 h under vacuum. The pre-cured PDMS film was cured at 120 °C for 1 h followed by additional curing at 150 °C for another 1 h. Unreacted elastomers and solvent were removed by successive rounds of ultrasonic cleaning of the cured film in acetone, isopropanol and distilled water, respectively, for 30 min per wash. This cleaning step was repeated two more times.

To synthesize AgNPs on the the PDMS films, the films underwent air plasma treatment in a 18W plasma cleaner (PDC-32G-2, Harrick Plasma) for 10 min under vacuum. To form Ag seeds on the film, immediately after the plasma treatment the PDMS films were immersed in 150 ml AgNO3 (10 mM) solution at room temperature for 1 h, followed by the addition of 3 ml NaBH_4_ (50 mM) solution and incubation for 15 min. To grow Ag seeds, the PDMS film in 150 ml 0.0167% AgNO_3_ was heated to 80 °C, followed by the injection of 3 ml 1% (by weight) sodium citrate (Sigma-Aldrich) as a reducing agent of silver ions. After injecting sodium citrate, the color of the solution that contained the PDMS film turned to brown within 5 min. The AgNP growth reaction was incubated for 2 h and repeated once or twice as needed. Residues on the film, such as the organic solvent from the elastomers, were removed by ultrasonic cleaning, as described above. The formation of AgNPs on the PDMS films was monitored by measuring the absorbance spectra of the film with a UV–Vis–NIR spectrometer (Agilent Technologies, Cary 5000). The size and shape of the AgNPs were investigated by imaging the films using a scattering emission microscope (Hitachi SU8010). The amount of AgNPs on the film was analyzed with an inductively coupled plasma mass spectrometer (Agilent ICP-MS 7700S).

To determine the optimal number of rounds of the AgNP growth reaction, antibacterial activity was determined by spraying *E. coli* cultures on a PDMS film and measuring the numbers of colonies on the film [14]. First, a 100 ml aliquot of a bacterial suspension in PBS (~ 10^11^ cells per ml) that had been stored at − 20 °C was thawed at room temperature. The bacterial suspension was added to a sterile 50 ml centrifuge tube that contained 20 ml yeast dextrose broth and was shaken at 200 rpm at 20 °C overnight. The next day, the bacterial suspension was centrifuged at 6000 rpm (11,470×*g*) at room temperature for 10 min, and the bacterial pellet was resuspended in 20 ml PBS and vortexed for 2 min; this step was repeated once more. The harvested bacterial cells were diluted in PBS to a final concentration of 5 × 10^7^ cells per ml by measuring the OD of the bacterial suspension at 540 nm (an OD value of ~ 1 indicates 10^9^ bacterial cells per ml). The diluted bacterial suspensions were sprayed onto a PDMS film at a rate of ~ 10 ml min^−1^ by adjusting the air flow with a chromatography sprayer. Films were air dried for 2 min at room temperature and placed in a Petri dish covered with solid growth agar (25 × 75 mm^2^) followed by incubation of the Petri dish after sealing with Parafilm at 37 °C overnight. Colonies that had grown on the tested film were then compared. The reduction in bacterial growth by the AgNP-grown PDMS film was estimated according to a modified version of ISO 22196, a protocol for measuring the antimicrobial activity of plastic surfaces [15]. A total of 10 ml MHB was inoculated and cultured overnight at 37 °C in a shaker. On the day of the test, 200 μl of the overnight culture was grown in 10 ml fresh MHB and shaken at 37 °C for 1 h to achieve exponential growth. The 1 h culture was diluted with saline using a spectrophotometer to achieve a cell density of 10^7^ colony forming units (CFU) ml^−1^. Each film surface was inoculated with 50 μl 10^7^ CFU ml^−1^ culture and covered with a polyethylene film to prevent drying of the inoculated culture. Each film was incubated at room temperature in a Petri dish sealed with Parafilm containing pieces of paper saturated with water to avoid drying of the test surface. Bacterial growth after 24 h of inoculation was determined by transferring the film to a 50-ml conical tube filled with 10 ml 0.9% sodium chloride solution, which was stirred for 30 s to recover the inoculum. Finally, 1 ml of the resulting solution was serially diluted, plated on Mueller–Hinton agar, and incubated at 37 °C for 24 h to determine the final CFU. The log_10_ reduction was calculated from the initial inoculum by subtracting the log_10_ count from the control assay on the test surface.

Biological safety of the AgNP-fabricated on PDMS film was examined with the MTT assay using the film elute and direct contact of the film to the tested cells (fibroblast cells). The MTT assay was carried out according to ISO 1093-5 [16]. The PDMS film elute was prepared by immersing the PDMS film (25 × 75 mm^2^) in 20 ml EMEM that contained 10% FBS and 1× antibiotic. Cells were exposed to the material elute or allowed to contact sterilized PDMS film (10 × 10 mm^2^) for 24 h. For the control group, 10^5^ cells were cultured for 24 h, and then were cultured in fresh EMEM without serum for another 24 h. Cell viability was determined using the MTT assay. The MTT solution was prepared at 1 mg ml^−1^ in complete medium immediately prior to use. Then, 0.6 ml MTT solution was added to each well, and cells were incubated in a humidified atmosphere of 5% CO_2_ at 37 °C for 4 h. After the incubation period, supernatant was removed and dark blue formazan crystals that had formed were dissolved in 0.6 ml ethanol. Plates were shaken for 5 min and then the blue solution was transferred to a 96-well plate; optical densities were read at 570 nm in a multiwell spectrophotometer (TECAN Infinite® 200).

## Results and discussion

**Fig. 1:**
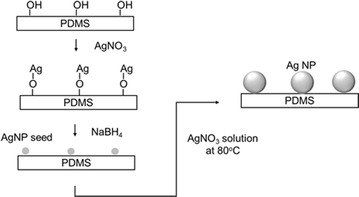
Schematic illustration of the in situ synthesis of AgNPs on PDMS film

**Fig. 2:**
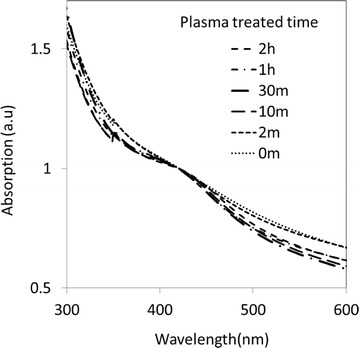
Absorption spectra of AgNP seed-fabricated PDMS films subjected to various times of plasma treatment

The process used in the in situ synthesis of AgNPs on PDMS surfaces is illustrated in Fig. 1. AgNPs are easily formed by the reduction of silver ions with reducing agents (e.g., NaBH_4_, hydroquinone, or sodium citrate) [17]. To form AgNPs attached the polymer surface instead of in a solution, we introduced hydroxyl groups on the surface because this functional group has been used as anchors for the adsorption of positive metal ions [18, 19] and can be easily formed by exposure to an air plasma [20, 21]. Figure 2 shows the spectra of the polymer films with different plasma exposure time for reactions with NaBH_4_ to form AgNPs on the surface. AgNPs, ranging from 10 to 40 nm, have a typical plasmonic absorption spectrum with a maximum peak around 400 nm. As shown in Fig. 2, the polymer films exposed to plasma for more than or equal to 10 min have maximum absorption peaks around 400 nm, which indicates the formation of AgNPs. However, no difference of absorption spectrum was observed between the control film and film treated with plasma for less than 10 min. This analysis of the absorption spectrum supports the role of hydroxyl group in capturing silver ions for the in situ formation of AgNPs. Since those PDMS films exposed to plasma for more than 10 min show no significant changes of the absorption, we used the PDMS films exposed to the air plasma for 10 min for further investigations.

**Fig. 3:**
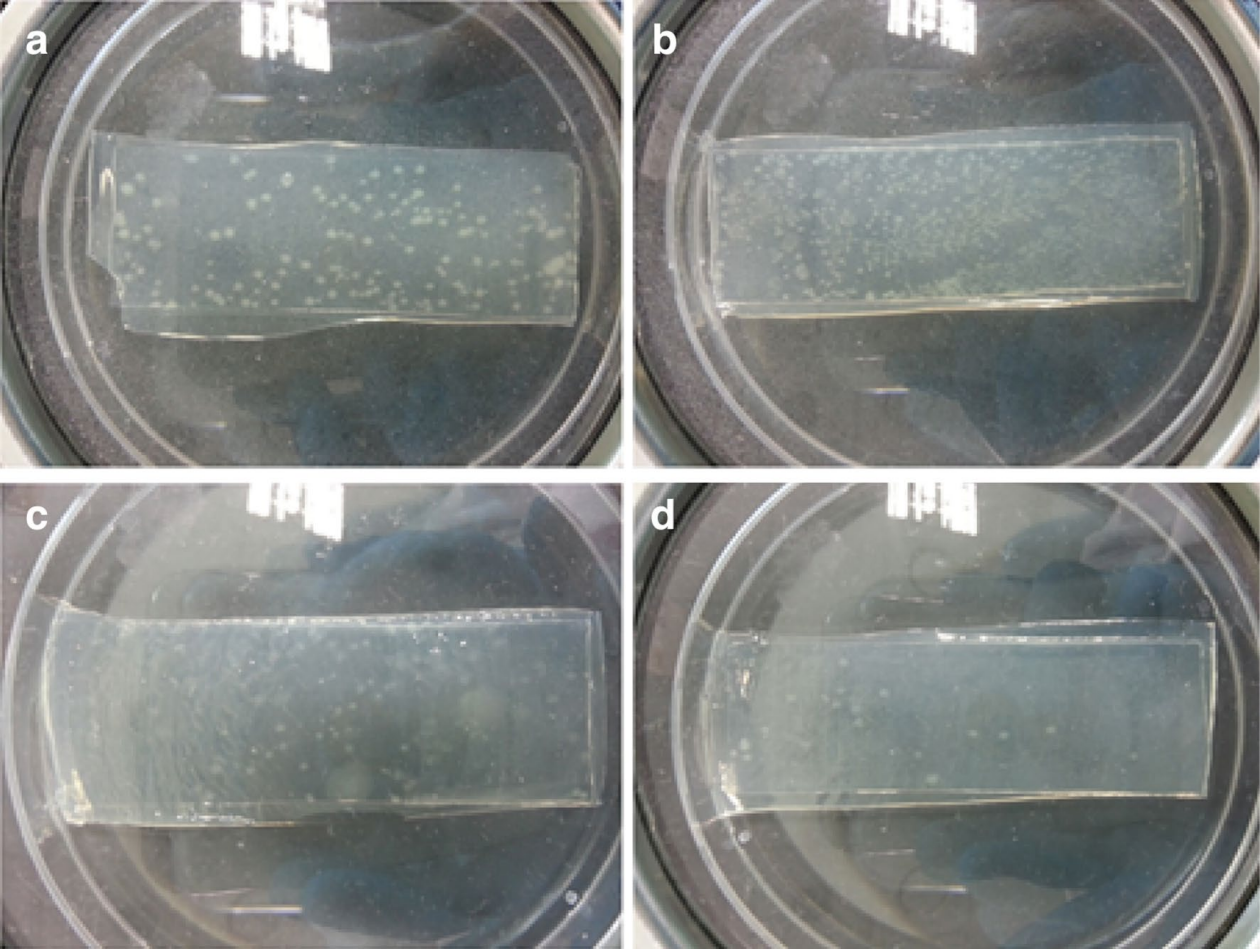
Effect of organic solvent residues and plasmon treatment time on the antibacterial activity of PDMS films. **a** Uncleaned and **b** cleaned PDMS films before the AgNP fabrication. **c** Plasma untreated and **d** treated PDMS films with AgNP seeds on the film

In this study, all the PDMS films were fabricated with elastomers that contained xylene. Therefore, we recognized a need to investigate any growth inhibition of bacteria by residues of organic solvents for the elastomers on the polymer film. To remove xylene residues from the polymer film, we cleaned the PDMS film according to the manufacturer’s protocol with successive washes of acetone and ethanol. Any growth inhibition of bacteria attributed to the organic solvent was estimated by comparing the number of colonies formed on the solvent-cleaned films and uncleaned control film at 24 h after spraying *E. coli* cultures on the films. Figure 3a, b show that more colonies were formed on the controls than on the solvent-cleaned film. This result indicates that the organic solvent residue could interfere with AgNPs in assessments of the inhibition of bacterial growth. Furthermore, the organic solvent is potentially hazardous for clinical applications. However, we removed the xylene residue on the polymer film for all tests of antibacterial activity and biosafety. We estimated the antibacterial activity of the AgNO_3_-reduced PDMS films by comparing the number of colonies formed on the film with that on a control film that was prepared without the AgNO_2_ reducing step. As shown in Fig. 3c, d, fewer bacterial colonies were observed on the AgNO_3_-reduced films (~ 40 colonies) than on the control films (~ 90 colonies).

**Fig. 4:**
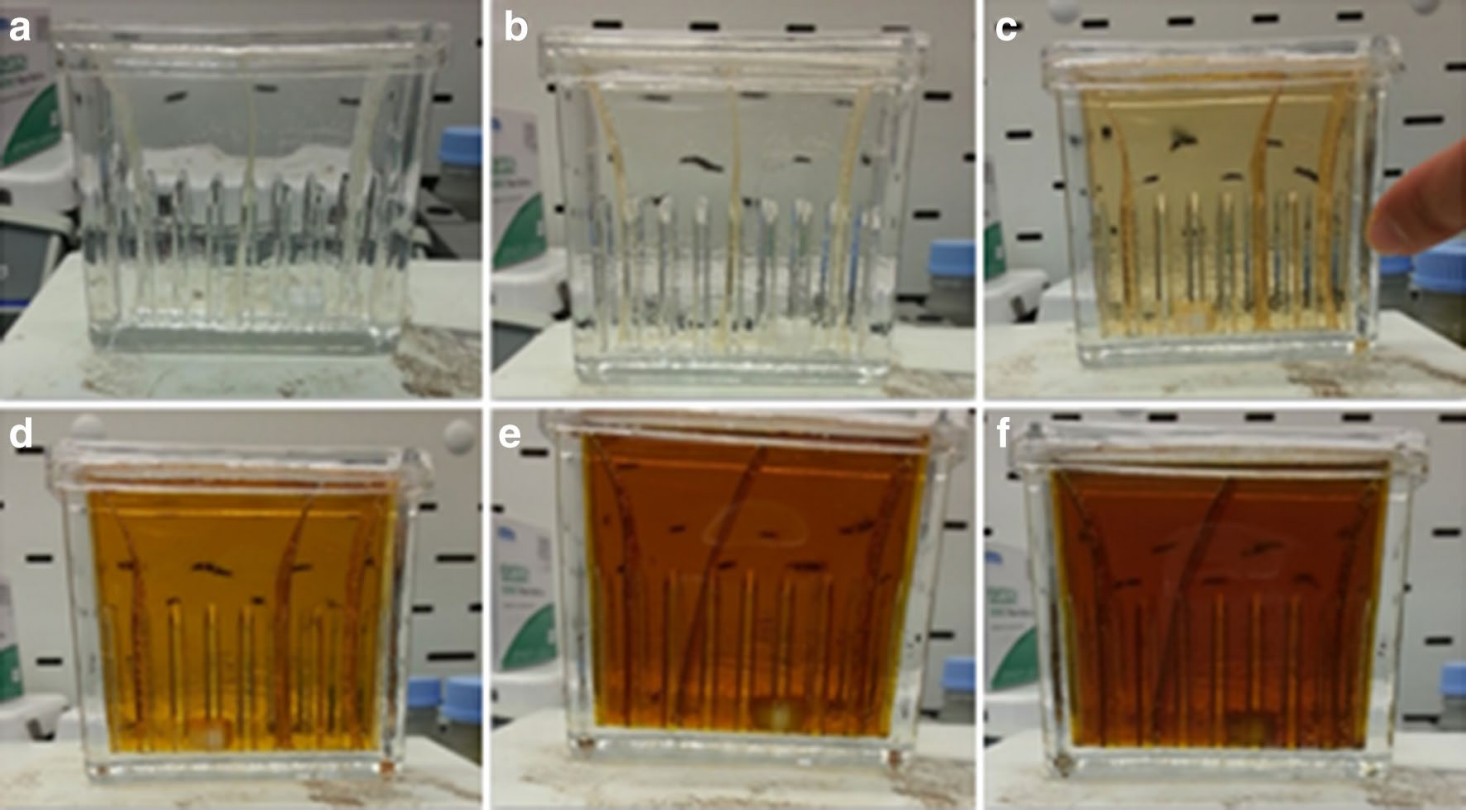
Time-dependence of the AgNP growing reaction on PDMS film in a solution that contained AgNPs at 80 °C after the addition of sodium citrate. **a** 1 min, **b** 5 min, **c** 10 min, **d** 15 min, **e** 25 min, and **f** 35 min

**Fig. 5:**
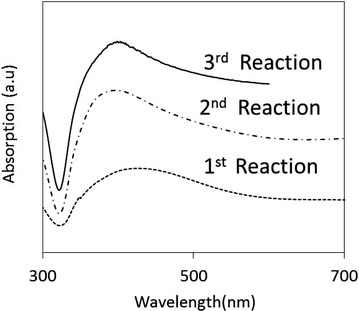
Absorption spectra of PDMS films depending on the number of rounds of the additional AgNP-growth reaction

**Fig. 6:**
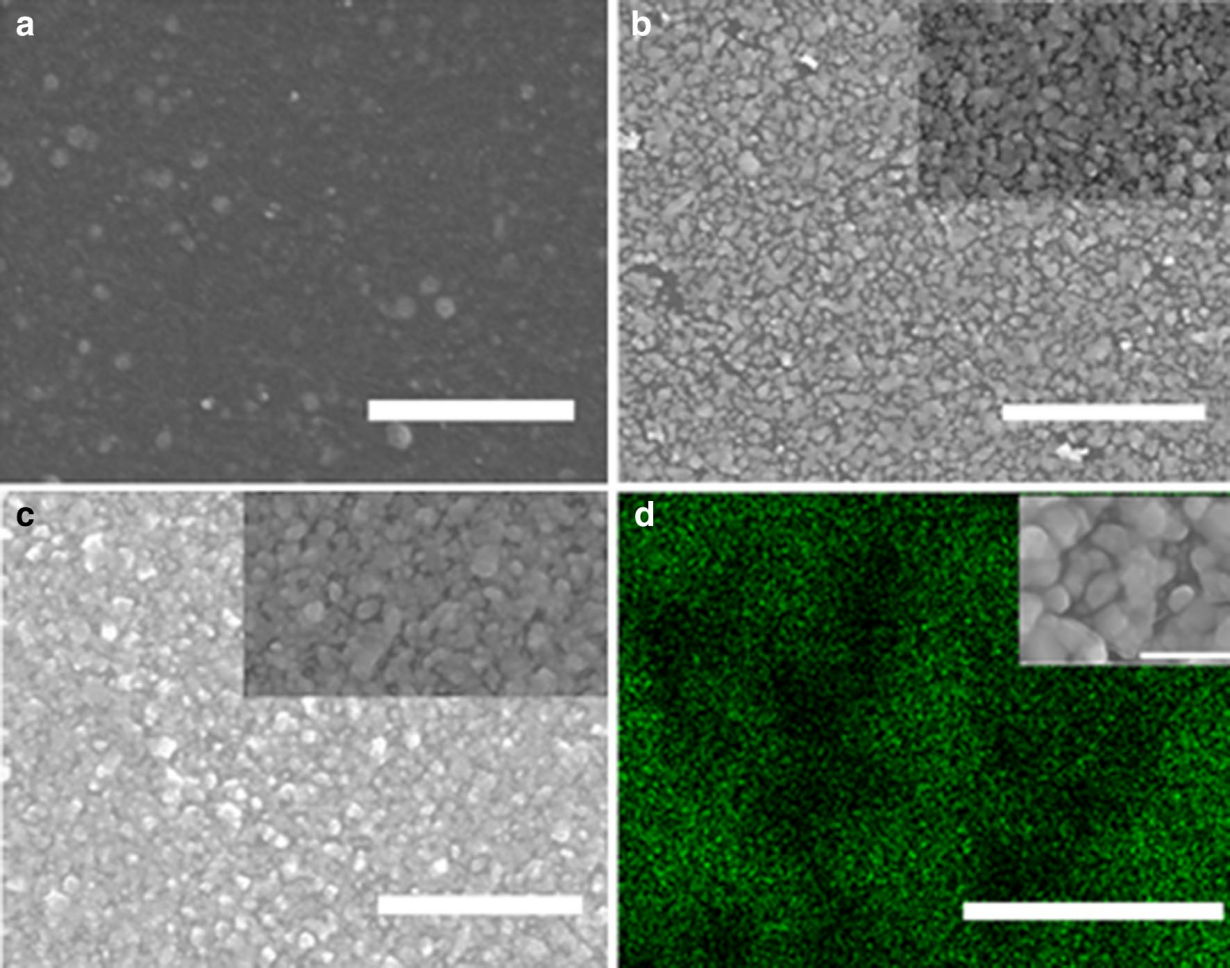
Electron microscopic analysis of the AgNP-grown PDMS films subjected to various rounds of reactions. From **a** to **c**; one, two and three rounds of reaction, respectively. **d** Ion mapping image and inset of **d** corresponding electron microscopic images of AgNP-grown PDMS films after three rounds of the AgNP growing reaction. Insets in **b** and **c** are enlarged images of the figures. 500 nm scale bar in each SEM image

Notably, the FDA requires at least a four log_10_ reduction of bacteria for medical devices to be classified as showing antibacterial activity. Since we inferred that the amount of AgNPs on the film was not sufficient to adequately inhibit the growth of *E. coli*, additional growth reaction of AgNPs on the formed film was carried out. Figure 4 shows time-resolved images of the process used to promote the additional growth of AgNPs on the PDMS films in a solution that contained AgNO_3_ and sodium citrate as reducing agents. After 10 min, the color of the AgNO_3_-containing solution clearly turned to brown, which indicated the formation of AgNPs in the solution. As the reaction time proceeded, the color of the solution became darker (Fig. 4). The growth of AgNPs on the polymer films was investigated by measuring absorption spectra and obtaining electron microscopic images of the polymer films. Figure 5 shows the absorption spectra of PDMS films with a typical plasmonic absorption spectrum of AgNPs after the additional growth of AgNPs. Compared with the results presented in Fig. 1, the significantly increased plasmonic absorption peak of AgNPs indicates that an increased portion of AgNPs was present on the film. There was a distinct color change of the PDMS film from a colorless transparent surface to a mirror-like surface with either silver or yellow color, depending upon the viewing angle. We carried out this reaction two more times. As the number of reactions increased, the plasmonic peak of the AgNPs increased and the color of the PDMS film darkened. Figure 6 shows scanning microscopic images of PDMS films subjected to different numbers of reactions. The SEM image of the PDS film subjected to one reaction reveals that mostly spherical silver nanostructures had formed. After two or three rounds of reactions, the nanostructures became big and irregular shapes that occupied most of film surface. The matched outline between the SEMS and ion mapping images confirmed the composition of the nanostructure as silver ions. We performed ICP–MS analysis of the AgNP on the film. Of the total weight of film, 0.048% was accounted for by silver ions.

**Fig. 7:**
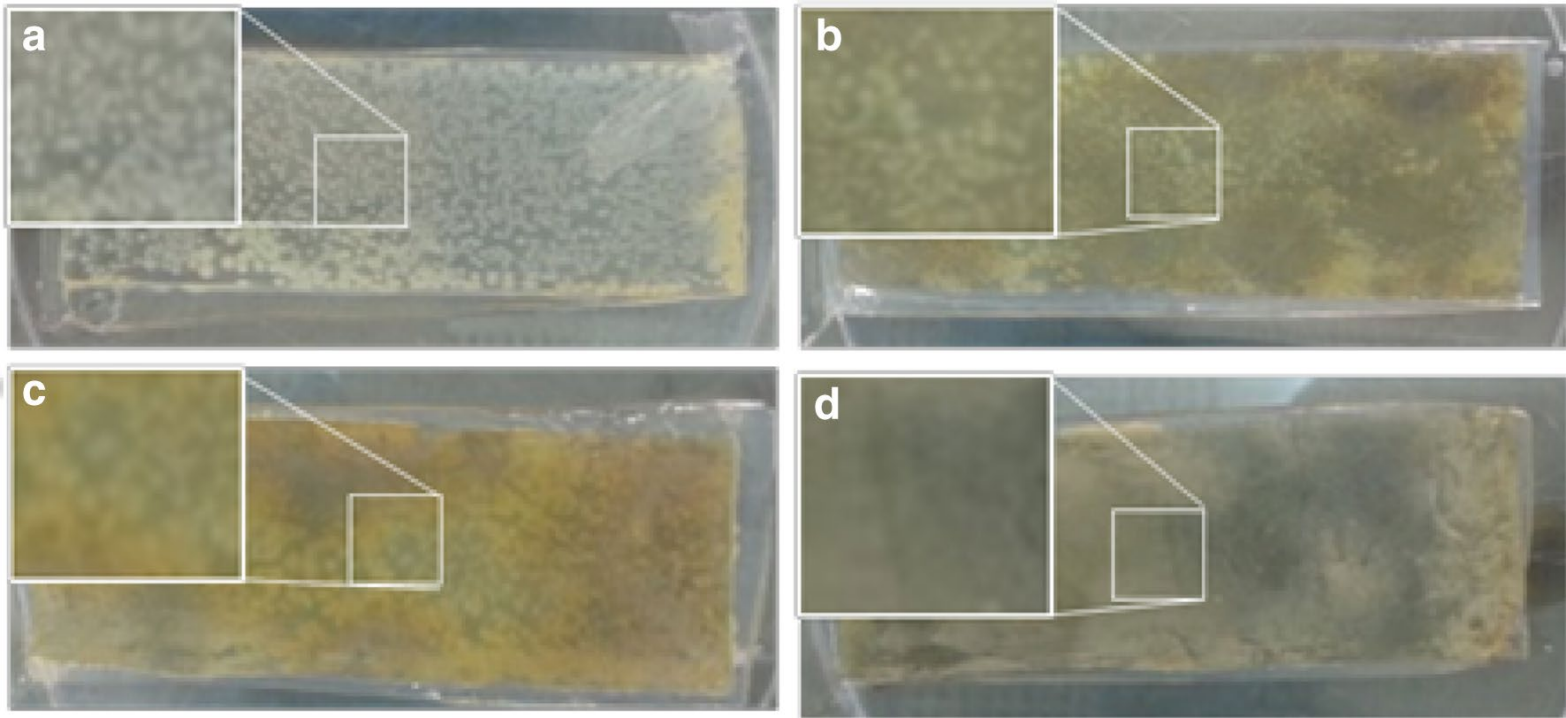
Photographic images of an AgNP grown PDMS film at 36 h after inoculation of S*. aureus* on the surface. **a** Control PDMS was prepared without the AgNP growing reaction. From **b** to **d**, AgNP-grown PDMS films subjected to one, two, or three rounds of reactions, respectively. Inset in each figure is an enlarged image of the figure

Before estimating the antibacterial effectiveness, we screened to identify the optimal number of reactions using *S. aureus*. As shown in Fig. 7 no colony formation was observed on the PDMS films that were reacted for three rounds, suggesting that this treatment resulted in the greatest antibacterial activity.

**Fig. 8:**
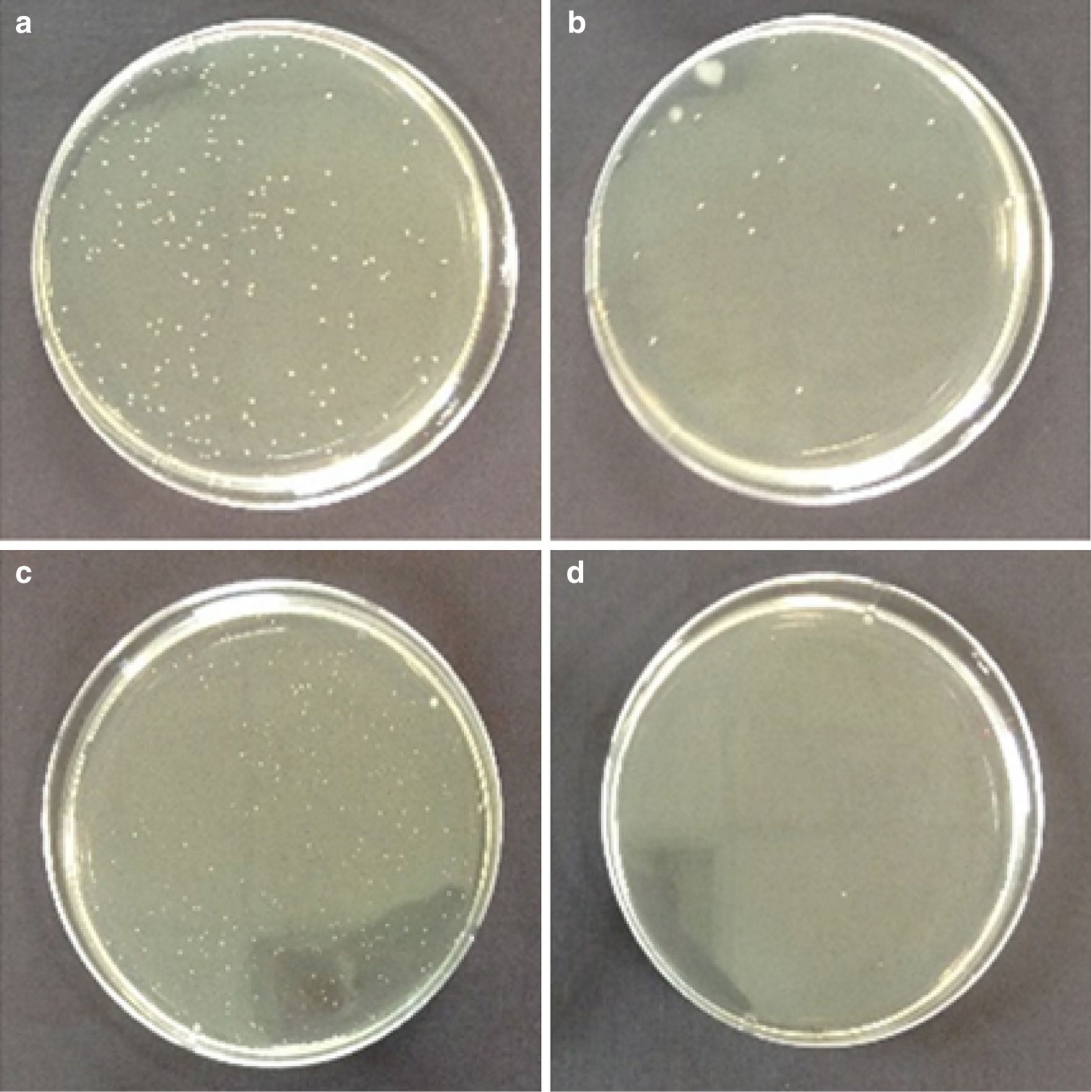
Colony formations on Mueller–Hinton agar inoculated with diluted solution of the collected elution from the bacterial inoculum on the AgNP-grown PDMS films. **a**, **b** *E. coli* inoculum of 1 × 10^5^ dilution of the control and 1 × 10^3^ dilution of AgNP-grown PDMS film o the agar, respectively. **c**, **d** *S. aureus* inoculum of 1 × 10^4^ dilution of the control and 1 × dilution of AgNP-grown PDMS films, respectively

**Fig. 9:**
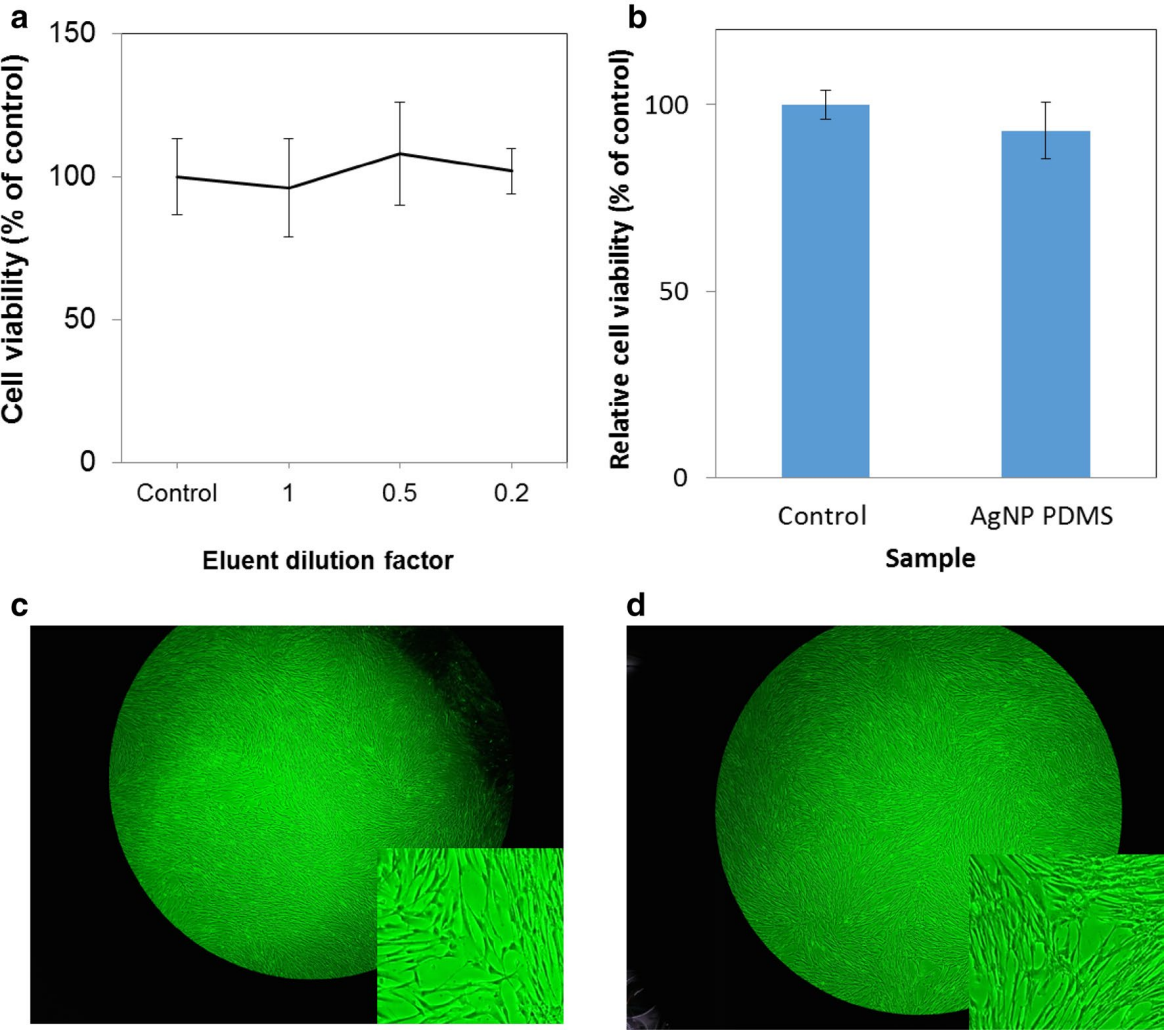
Cytotoxicity tested with **a** eluent and **b** direct contact of the AgNP-grown PDMS film. Microscopic images of the tested cells grown in direct contact with **c** control PDMS film and **d** AgNP-grown PDMS films. Inset of **c** and **d** is enlarged image of each figure

**Fig. 10:**
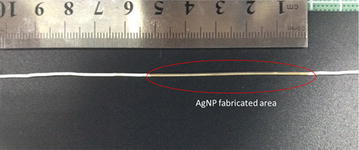
Photographic image of an in situ AgNP fabricated catheter for use in a pre-clinical test

Therefore, we determined the order of bacterial reduction using the PDMS films subjected to three rounds of reaction based on a method described in ISO 22913. According to this method, the number of colonies after 24 h incubation with *E. coli* and *S. aureus* that were applied separately to the PDMS film covered with polyethylene film was counted and compared with that on a control film without AgNPs (Fig. 8). The measured bacterial reduction on the PDMS films was log_10_ 4.8 and log_10_ 5.7 for *E. coli* and *S. aureus*, respectively. The in situ AgNP grown PDMS films demonstrated greater antibacterial activity than the requirement of FDA. Additionally, in order to be used as an implantable medical device, the biological safety of the devices should be guaranteed. We examined the biological safety of the PDMS film by the MTT cytotoxicity test using an eluent of the AgNP-grown PDMS film and direct contact of the films on test cells. As shown in Fig. 9, no significant AgNP-mediated cytotoxicity driven by AgNP on PDMS film was observed. We also examined cell morphology after this treatment. Those cells that contacted the AgNP-grown PDMS films had an identical morphology to the reference cells. These two findings confirmed the biological safety of AgNP-grown PDMS film, as prepared according to the conditions described in this study. Finally, we could fabricate successfully AgNP-attached catheter by applying this in situ method for a preclinical test of the catheters as an antibacterial implantable medical device (Fig. 10). The same color as the AgNP synthesized silicone films indicates formation of AgNP on the treated area of the catheter. Since the catheter was made of silicone rubber powders, the same procedure without any modification as used for the silicon films was applied for the in situ formation of AgNPs on the catheter.

## Conclusion

In the present study, we successfully demonstrated the in situ synthesis of AgNPs on the PDMS film for use as an antibacterial surface. The AgNP–PDMS composite showed high antibacterial activity against representative infectious bacterial growth. Of the total weight of the film, a small amount was represented by silver. Since all of the AgNPs were placed on the surface of PDMS film, they (~ only 0.048% of the total weigh of the PDMS film) could act to prevent bacterial growth, resulting in effective antibacterial activity. The oxidative release of Ag^+^ from Ag^0^ NP is known to drive antibacterial actions, such as binding to a wide variety of targets that contain organic amines, phosphates, thiol-containing proteins, and DNA, and can increase reactive oxygen species and simultaneously perturb many aspects of the cell metabolism [6]. Thus, the release of silver ions can negatively impact the surrounding cells via similar antibacterial actions. Therefore, it is crucial to minimize the cytotoxicity of the antibacterial surface while maintaining its bacterial killing activity. There are several ways to avoid the side effects of AgNPs for uses in medical applications, such as the layer-by-layer technique [22] and plasma immersion ion implantation [23], to prepare AgNP-containing antibacterial polymer films. In a comparison of these methods, the in situ synthesis of AgNPs on the PDMS film presented herein is straightforward and less dependent on instruments. Consequently, it could be easily integrated in the existing manufacturing process of PDMS-based medical devices. Goyal et al. reported an in situ synthesis of AgNP inside of the PDMS film by mixing silver precursors with elastomers and then reducing them [13]. Since most AgNPs were embedded in the film, only 80 and 52% reduction growth of *E. coli* and *B. subtilis*, respectively, was reported. By contrast, we achieved more than a five log_10_ reduction of bacteria with a small amount of AgNPs because all AgNPs synthesized in present study were located on the film surface. We believe that our finding could be useful for manufacturing a silicone-based implanting device with antibacterial activity by proving the straightforward and price economically competitive method.
